# Management alternatives for *Carmenta theobromae* (Busck, 1910) (Lepidoptera: Sesiidae) and *Simplicivalva ampliophilobia* (Lepidoptera: Cossidae), limiting pests of guava in Colombia

**DOI:** 10.1038/s41598-021-81830-3

**Published:** 2021-02-04

**Authors:** Víctor Camilo Pulido-Blanco, Elberth Hernando Pinzón-Sandoval, Carlos Felipe González-Chavarro, Pablo Antonio Serrano-Cely

**Affiliations:** 1 Centro de Investigación Tibaitatá Sede Tunja, MSc. Ciencias Biológicas, Corporación Colombiana de Investigación Agropecuaria - AGROSAVIA, Kilometro 14 vía Mosquera, Cundinamarca, Colombia; 2 Calle 19 N° 9-35 Edificio de la Lotería de Boyacá, oficina 902, Tunja, Boyacá Colombia; 3 Facultad de Ciencias Agropecuarias, MSc. Fisiología Vegetal, Universidad Pedagógica y Tecnológica de Colombia, Tunja, Colombia; 4 Centro de Investigación La Libertad, MSc. Fisiología Vegetal, Corporación Colombiana de Investigación Agropecuaria - AGROSAVIA, Kilometro 17 vía Puerto López, Meta, Colombia; 5 Facultad de Ciencias Agropecuarias, MSc Ciencias Ambientales, Universidad Pedagógica y Tecnológica de Colombia, Tunja, Colombia

**Keywords:** Entomology, Plant sciences

## Abstract

The larval stages of *Carmenta theobromae* Busck (1910) and *Simplicivalva ampliophilobia* Davis, Gentili-Poole and Mitter (2008) attack the subcortical zone and pith in guava trees, respectively, in the first productive nucleus of fruit trees in Colombia: Hoya del Río Suárez (HRS). The presence of pest insects has been reported in 98% of the farms sampled in HRS (n = 124), with up to 96 and 11 simultaneous larvae per tree, respectively. Although the aspects of the basic biology and life cycle of both pests have been resolved, there are no strategies for managing populations in the field. Therefore, the aim of this study was to evaluate different management alternatives under laboratory and field conditions in HRS. In laboratory conditions, a completely randomized design was used in two separate experiments, each with six treatments: T1: Spinosad (a mixture of Spinosad A and D); T2: S-1,2-di(ethoxycarbonyl) ethyl 0,0-dimethylphosphorodithioate (chemical control); T3: *Lecanicillium lecanii*; T4: *Beauveria bassiana*; T5: Mix of *B. bassiana* and *B. brongniartii*, and T6: distilled water (control). The number of dead larvae per replicate per treatment was evaluated (DL), with experimental units of five and three larvae, respectively. In the field, to the two best alternatives found for each pest in the laboratory, pruning and keeping the area around the plants free of weeds were added as cultural management, in two separate additional experiments, each with three larvae as experimental unit per treatment. For *C. theobromae,* the best laboratory alternatives were chemical control (DL: 3.78) and *L. lecanii* (DL: 2.33), followed without statistical differences by *B. bassiana* (DL: 1.67). In the field, the virulence of *B. bassiana* improved (DL: 3), and together with pruning and keeping the area around the plants clear of weeds (DL: 3), they stood out as the best alternatives. For *S. ampliophilobia* under laboratory conditions, the best alternatives were Spinosad (2.74) and chemical control (DL: 2.66), without significant difference. In the field, there were no statistical differences between the alternatives, except for the control. This statistical parity of cultural practices, and biological and chemical management is an argument in favor of the use of the former to the detriment of the third, especially when the harmful effects of the molecule S-1,2 di (ethoxycarbonyl) ethyl 0, 0-dimethyl phosphorodithioate have been proven in air, water and agricultural soils, in addition to its association with thyroid cancer in humans. This is a strong argument to favor the use of synergies of cultural and biological management methods framed in IPM, as opposed to the use of chemical agents whose harmful effects are strongly documented, and whose use is becoming increasingly prohibited.

## Introduction

The Sesiidae family (Boisduval, 1828) (Lepidoptera) is comprised of 151 genera and 1,370 species, including 50 subspecies^1^. Their larvae are of agricultural interest because they are xylophagous insects that bore live wood (stems, branches, and roots) from shrubby, arboreal, and herbaceous hosts^2^. Most research on this family focuses on the biological aspects of the << dogwood borer >>, larvae of the *Synanthedon* genus found in multiple plant hosts, especially timber species from the northern and southern temperate zones^3,4^. However, towards the tropical zone in recent decades, the presence of the genus *Carmenta*, with the species *C. foraseminis* and *C. theobromae*^5^, stands out. The larval stages of *C. theobromae* have been reported attacking mainly cacao fruits^5,6^, as well as the subcortical zone of guava trees^6,7^, and myrtle^6^ in Venezuela and Colombia.

The attack of *C. theobromae* is considered a limitation in the two largest guava production centers in Colombia: Hoya del Río Suárez (HRS) and the northern region of Valle del Cauca (NVC)^6^. The presence of the pest insect has been reported in 98% of the farms sampled in HRS (n = 124), with approximately 10 infested trees over 40 observed, and up to 96 simultaneous larvae per tree. In the NVC its presence has been registered in the north and center of the department, in technified systems of pear guava Palmira ICA-1 and wild plots of common guava, as well as in cacao plantations^6^.

Likewise, in the same hosts, a recently described species has been found, identified as *Simplicivalva ampliophilobia* Davis, Gentili-Poole and Mitter (2008)^8^. The larval stage of this species can reach 5 cm in length and has been observed drilling the stem pith and primary and secondary branches of guava trees in HRS. Reports indicate that it can induce significant damage to the attacked trees causing their death and has been considered to be more aggressive than *C. theobromae* and other common guava pests, with an average incidence of 96%, and a severe attack degree with up to 11 larvae per tree.

Even though the basic biology and life cycle aspects of both the guava bander *C. theobromae* and the guava borer *S. ampliophilobia* have already been studied^7,9^, as well as the natural enemies present in the guava nuclei of HRS and NVC, and some monitoring methods including a pheromone for the bander^5,10,11^, so far there are no reports on the evaluation of integrated management strategies for these two pests.

Integrated pest management (IPM) has been considered as a promising solution to problems caused by insects within a perspective of sustainable agriculture^12^. In Colombia, there are reports of successful cases of applying IPM for crop limiting insects in cassava^13^ and Cape gooseberry^14^. However, the adoption of IPM in Latin America is, in general, marginal^12^. One reason is the few positive results in field validations, especially when live biological management agents are involved. In this sense, it is necessary to obtain laboratory results that allow predicting the behavior of the management alternatives in their application under field conditions. With this, it is possible to focus efforts on the most promising alternatives, saving resources in their validation^15^. From this perspective, the aim of this study is to evaluate different management alternatives for *C. theobromae* and *S. ampliaphilobia,* both in the laboratory and in commercial guava fields in HRS, to identify the best alternatives. This seeks to provide a mediated response to a problem with relevance for the development of guava cultivation in Colombia.

## Materials and methods

Specimens were collected in silvo-pastoral guava plots located in HRS in the municipalities of Vélez, Jesús María, Guavatá, Puente Nacional, San Benito, Chipatá, Güepsa and Barbosa, in the department of Santander, and in the municipalities of Moniquirá, Provincia de Ricaurte, and Briceño and Tununguá in the western region of the department of Boyacá, Colombia. Silvo-pastoral and non-technified plots were chosen because they have high percentages of infestation of pest insects.

Laboratory studies were carried out at the agricultural entomology laboratory of the research center C.I. Tibaitatá of Agrosavia, Cimpa campus, located between 5°56′51″ N and 73°36′24″ W. The experimental work in the field was carried out in Vélez, department of Santander, in the sector Los Guayabos, district of Aco and Peña Blanca, in the farm El Paraíso located between 5°58′27″ N and 73°39′45″ W, and above 1905 m above the sea level.

**Figure 1:**
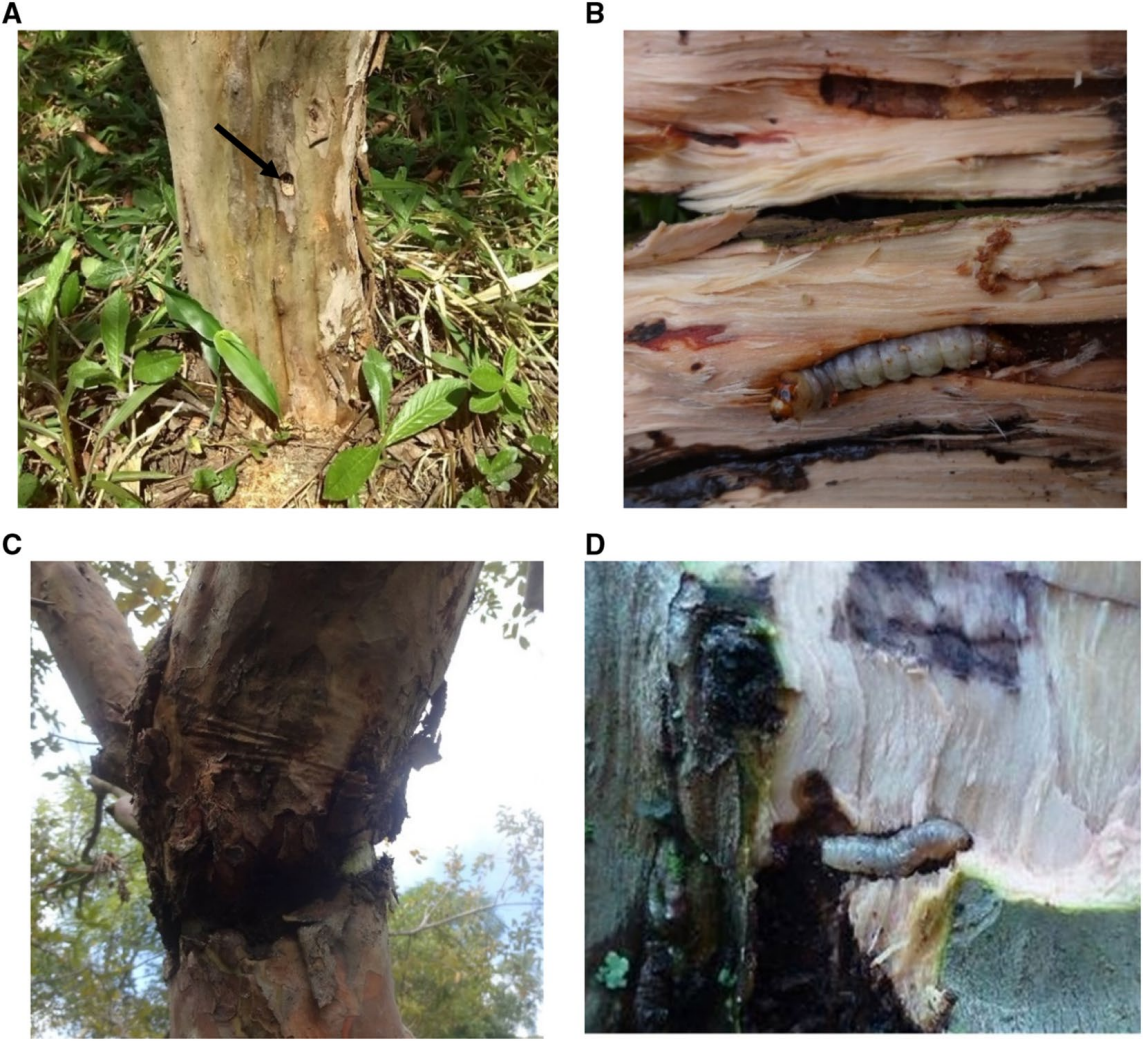
**a** Presence of *S. ampliophilobia* inside a guava tree due to observing a typical capped hole made by this insect. **b** *S. amphoraphilobia* larva inside a sectioned guava tree. **c** Presence of *C. theobromae*. **d** *C. theobromae* larva devouring the vascular cambium. *Source*: Pulido Blanco (2020).

Larvae were collected between March 27, 2015, and May 04, 2016, to obtain individuals from both pests for laboratory and field tests. Larva collections from I to VI instars were carried out through direct captures at sites showing recent activity (Fig. 1a,c,d). Pupae were also collected to obtain newborn larvae in the laboratory. All the captures imply the destruction of the guava tree to achieve the extraction of the individuals of *S. ampliophilobia* (Fig. 1 b).

The biological material (larvae and pupae) was transported in chambers conditioned at 22 °C, and 70% relative humidity (RH), and entered the laboratory emulating field conditions: 25 ± 3 °C and 60 ± 10% RH, with a photoperiod of 0:24. All the larvae were disinfected in a laminar flow cabinet, washing with 0.5% v/v NaClO for 5 s, followed by two washes with distilled and deionized water for 30 s.

The disinfected larvae were measured with an entomological ruler under a stereomicroscope and weighed with a Sartorius CPA3235 balance (d = 0.001 g) (supplementary Tables 1, 2, 3 and 4), for their subsequent distribution among treatments. Some larvae had to be immobilized with cold following the protocol of^16^. The total collections were recorded in a laboratory log and reported to the Alexander Von Humboldt institute through the Collection Permit of the Colombian Ministry of Environment and Sustainable Development. The larvae were deposited as follows: *C. theobromae* in a meridic diet of cacao prepared based^17,18^, varying the main ingredient, the antibiotic, the fat source, and the water concentration (Table 1). On the other hand, *S. ampliophilobia* was given a guava oligidic diet.

**Table 1:** Meridic diet for *Carmenta theobromae* proposed and used in this study.

Ingredients	Approximate amount to prepare		
	**4 kg**	**2 kg**	**1 kg**
Cocoa shell¨ (g)	800	400	200
Linseed oil (cm^3^)*	2	1	0.5
Methylparaben (g)	4	2	1
Agar–agar (g)	77	38.5	19.5
Distilled water (cm^3^)	3200	1600	800
Sorbic acid (g)	9.2	4.6	2.3
Ascorbic acid (g)	9.84	4.92	2.46
B complex (cm^3^)	0.736	0.368	0.184
Antibiotic (chloramphenicol) (g)	1.076	0.535	0.269
Distilled water (cm^3^)	307.6	153.8	76.9

¨: refers to the exocarp of the fruit of *Theobroma cacao* L. as the main ingredient. Values for the approximate amount of this ingredient to prepare, include the water that naturally comprises the exocarp. *It can be replaced by cholesterol or another fat source.

*Source*: Pulido Blanco (2020).

For each pest, a completely randomized block design was used, in which the block factor was the size of the larvae, and therefore, the age^19^ (supplementary tables 5, 6, 7, and 8). The response unit was the death event for each larva per repetition per treatment (DL); and the experimental unit, as well as the number of repetitions, varied for each pest in the laboratory and field according to larvae availability. In total, four separate experiments were carried out: two for each pest, one in the laboratory and one in the field. The experimental units differed in size, as noted in the Table 2.

**Table 2:** Experimental design.

Plague	Phase	Exp. U	Treatments	Reps	Total
*Carmenta theobromae*	Laboratory	5	6	2	60
	Field	3	5	6	90
*Simplicivalva ampliophilobia*	Laboratory	3	6	4	72
	Field	3	5	4	60

*Exp. U.*: experimental unit (number of larvae), *Reps*.: repetitions.

*Source*: Pulido Blanco (2020).

Management treatments were: T1: Spinosad (a mixture of Spinosad A and D); T2: S-1,2-di(ethoxycarbonyl) ethyl 0,0-dimethyl phosphorodithioate (chemical handling); T3: *Lecanicillium lecanii* (commercial strain (cp)); T4: *Beauveria bassiana* (cp); T5: Mix of *B. bassiana* and *B. brongniartii* (cp), and T6: distilled water (control). All treatments were sprayed to the drop formation point following the manufacturer's instructions^20–23^, using conventional atomizers, to a final volume of 250 cm^3^ (Table 3). Treatments T1 to T5 are commercial products readily available in the study areas. Every three days, observation and recording of dead individuals were made as a response variable.

**Table 3:** Doses of management treatments.

Alternative	Manufacturer's instructions	Dose measured at 250 cm^3^
Spinosad (a mixture of Spinosad A and D)^1^	1L product/1.5L Water (ratio 1: 1.5)	100 cm^3^ of product and 150 cm^3^ of water
S-1,2-di(ethoxycarbonyl) ethyl 0,0-dimethyl phosphorodithioate (chemical handling)	1L of product/4L of Water (ratio 1: 4)	50 cm^3^ of product and 200 cm^3^ of water
Mix of *B. bassiana* and *B. brongniartii* (cp)	200–400 g of product/200 L of water. We take an average of 300 g	0.375 g of product dissolved in 250 cm^3^ of water
*Lecanicillium lecanii*	500 g of product/200 L of water	0.625 g of product dissolved in 250 cm^3^ of water
*Beauveria bassiana*	200 cm^3^ of product/200L of water (ratio 1: 1000)	0.25 cm^3^ of product and 249, 75 cm^3^ of water
Distilled water	250 cm^3^ of water	250 cm^3^ of water

*Source*:^20–23^.

Based on the laboratory results, the two best alternatives were prioritized in the field, added to the chemical management and cultural management practices, including formation pruning and keeping the area around the plants clear of weeds, as well as the control. The age of the collected larvae, the position of the trees where the treatments were established, and the edge effect were blocked.

The data were analyzed through ANOVAs with Tukey's mean comparison test at a significance of 5%, verifying the assumptions of the model with the IBM SPSS Statics software, version 19.

## Results and discussion

### Collections

In total, 2313 biological states of *C. theobromae* were collected, corresponding to 2152 larvae between I and VI instars (93%) and 161 pupae (7%), and 441 biological states of the guava borer (*S. ampliophilobia*), corresponding to 239 larvae between the I and VI instars (54.2%), 200 pupae (45.35%), and two adults (sporadic capture) (0.45%). However, for both pests, high mortality was evidenced in larvae from I to III instars, as expected for Lepidoptera with a type II survival curve (r strategists), where the first instars are the most vulnerable^24^ (supplementary Tables 1, 2, 3, and 4). There was a higher decrease in biological states of *S. ampliophilobia* compared to those observed for *C. theobromae*, due to the lack of overlap of biological states and a very long univoltine life cycle with only one generation per year.

High concentrations of individuals per tree delimited the focal nature of the pests, which could be due to the reduction of habitats suitable for their survival. Individuals of *C. theobromae* and *S. ampliophilobia* were not collected in western Boyacá, but unidentified Sesiidae and Buprestidae individuals were collected. This demonstrates that trophic niches, whenever available, will be occupied by species with trophic analogies^25^.

### Laboratory management alternatives

All model assumptions were achieved in the laboratory experiments. There are statistically significant differences between the management alternatives of *C. theobromae* and *S. ampliophilobia* under laboratory conditions after 37 and 42 days of evaluation of post-application dead larvae, respectively (n = 160; *p* value = 0.000; < α = 0.05, and n = 60; *p* value = 0.0001; α = 0.05, respectively) (supplementary Fig. 1 ).

**Figure 2:**
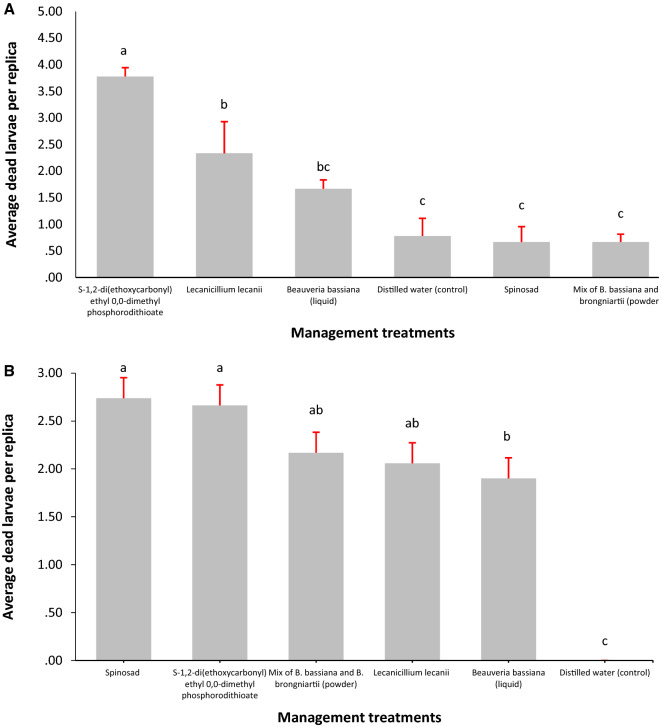
Average number of dead larvae per replicate under laboratory conditions. Different letters indicate statistically significant differences. **a** *C. theobromae* and **b** *S. ampliophilobia*. *Source*: Elaborated by the authors.

For *C. theobromae*, the chemical treatment S-1,2-di(ethoxycarbonyl) ethyl 0,0-dimethyl phosphorodithioate registered the highest average dead larvae per replica (3.78), followed, with statistically significant differences, by the treatment with the *L. lecanii* fungus (2.33). The treatment of *B. bassiana* in a liquid vehicle (1.67) was statistically different from the chemical control, but not to the *L. lecanii* fungus (Fig. 2a). The Spinosad and mix of *B. bassiana* and *B. brongniartii* powder treatments did not have statistically significant differences with the control and were discarded for field evaluation. The evaluation of management alternatives for *S. ampliophilobia* showed an early response in terms of the number of dead larvae per experimental unit: between the second and sixth days, with a new larval death event between the tenth and sixteenth days. The death events after these periods were verified under laboratory conditions due to starvation because of inappetence. Therefore, the recommendation is to avoid an evaluation that extends beyond 20 days of follow-up. The best treatment was Spinosad (2.74), followed, without statistical differences by the chemical treatment (2.66). The mixture treatment of *B. bassiana* and *B. brongniartii* powder and the one with *L. lecanii* did not show statistically significant differences between them (Fig. 2b). The treatment of *B. bassiana* in a liquid vehicle yielded the least number of dead *S. amphiophilobia* larvae among the alternatives, regardless of the control, which behaved as expected, without causing death events.

The chemical treatment S-1,2-di(ethoxycarbonyl) ethyl 0,0-dimethyl phosphorodithioate is widely used in Colombia, especially in HRS, for the effective and cheap control of the guava weevil *Conotrachelus psidii* Marshall (Coleoptera: Curculionidae), that is the main pest of the guava fruit tree, and a real limiting factor in *bocadillo* production (a popular snack in Colombia)^26^. Although the results on *C. theobromae* reinforce the thesis that the use of chemical synthesis insecticides persists due to their effectiveness^27–29^, toxic effects of S-1,2-di(ethoxycarbonyl) ethyl 0,0-dimethyl phosphorodithioate has been demonstrated by bioaccumulation in animal and plant species such as the common river carp *Cyprinus carpio carpio*^30^, cucumber^31^, and in native microorganism species in Colombian soils^32^. This molecule has been shown to pollute surface water, groundwater, soil, and even air persistently with few applications^33^. Its effects are not restricted to the organisms of the cropping field; its association with the increased risk of thyroid cancer in humans has been demonstrated^34^. Therefore, its use in fruit pest management programs has been restricted^35,36^. The results on *S. ampliophilobia* contribute to this trend, showing that an alternative such as Spinosad, a molecule of organic origin, has better experimental results than the chemical synthesis molecule most widely used in HRS.

The product based on the Spinosad molecule is registered by^37^ for the control of populations of Tephritidae in guava^26^, mango, and coffee in the HRS; furthermore, the molecule is globally recognized as a serious alternative to the control of this same family in a spectrum of tropical fruit trees with commercial interest^38,39^. Therefore, its use would not only contribute to the control of *S. ampliophilobia* populations but simultaneously to the management of fruit flies, especially the *Anastrepha* spp. complex and the Mediterranean fly *Ceratitis capitata*^26^.

Treatment with *B. bassiana* did not show the degree of effectiveness expected in the laboratory, which for Sesiidae related to *C. theobromae* has reached up to 76% of control, as in the case of *Synanthedon myopaeformis* in apple^40,41^. This may be due to the high specificity shown by some pathosystems, where the host exerts selection pressure on the genotypes of the pathogen, leading to the formation of host-specific forms^42^.

### Field management alternatives

**Figure 3:**
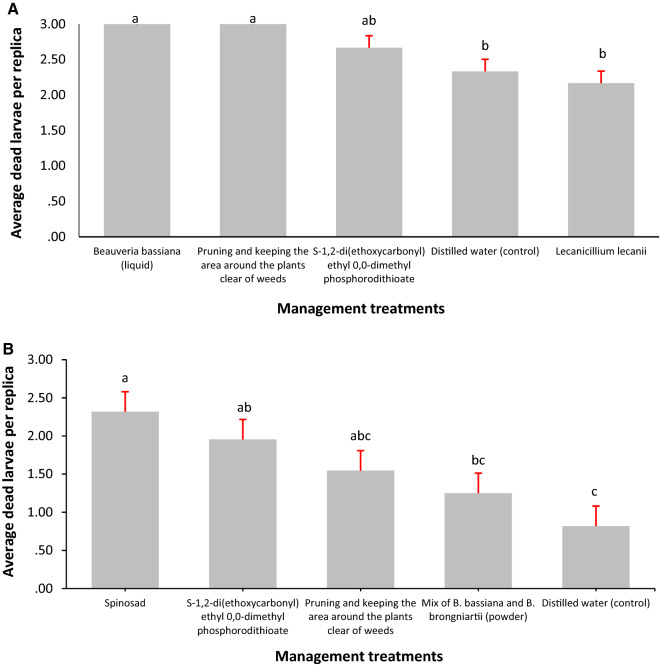
Average number of dead larvae per replicate under field conditions. Different letters indicate statistically significant differences. **a** *C. theobromae*. **b** *S. ampliophilobia*. *Source*: Elaborated by the authors.

All model assumptions were achieved in the field experiments. There are statistically significant differences between the management alternatives of *C. theobromae* (Fig. 3a) and *S. ampliophilobia* (Fig. 3b) under field conditions after 19 and 22 days of evaluation of dead larvae after spray inoculation, respectively (n = 90; *p* value = 0.013; < α = 0.05, and n = 60; *p* value = 0.000; α = 0.05, respectively) (supplementary Fig. 2). However, the differences in *S. ampliophilobia* occur between the alternatives and the control.

For *C. theobromae, B. bassiana* in a liquid vehicle and the cultural treatment that included pruning and keeping the area around the plants clear of weeds, registered the highest average dead larvae per replica after aspersion (3), without having statistically significant differences with the chemical treatment (2.67) (Fig. 3a). This situation reflects that the use of entomopathogenic fungi, in combination with adverse conditions for the establishment of pests, is an alternative that competes with the effects of chemical synthesis pesticides^43^. Treatment with the fungus *L. lecanii* did not have statistically significant differences with water.

Modifying the habitat of pests through pruning is positioned as a successful practice included in good agricultural practices^44,45^ point out that in Venezuela, the combination of pruning with the release of biological controllers is a frequent practice in approximately 6% of the guava producers in the country, while^46^ demonstrated that the combination with entomopathogenic fungi had a synergistic effect that potentiated the lethality of those formulated against *Premnotrypes vorax* larvae in potato. However, the study of cultural methods associated with entomopathogenic fungi has focused more on the virulence of the strains used than on the discernment of the mechanisms that allow synergies. This treatment bases its actions on the affectation of the two main abiotic variables that insects require to live, especially in the egg and larva stages: temperature and humidity^24^. Pruning increases the temperature and reduces humidity in the branches and stems that are exposed to sunlight^47^, while pruning and keeping the area around the plants clear of weeds eliminates sheltered areas near the stem. This causes unfavorable conditions for the oviposition of insects, which depends on the choice of a host that meets the necessary conditions to ensure the larvae cycle^48–52^. Thus, this treatment continuously affects the establishment of a pest near its substrate.

Entomopathogenic fungi showed a dual behavior in their passage from laboratory to field: *B. bassiana* improved its effectiveness, while *L. lecanii* decreased it. The improvement of *B. bassiana* agrees with^46,53^ who observed efficiency of *B. bassiana* isolates in the control of *Premnotrypes vorax*, both in vitro and in field conditions. However, contrasts with the results reported by^54^. These authors recorded a decrease in the virulence of their own *B. bassiana* strains on *Hypothenemus hampei* selected in the laboratory and evaluated in the field. Although^54^ recognized that the interactions of the entomopathogen and the host with the environment are unknown, in terms of control, these can occur in a two-way process: both negative and positive^55^. Just as environmental factors can inhibit the pathogenicity of entomopathogenic fungi, as happened with the mix of *B. bassiana* and *B. brongniartii* on *S. ampliophilobia*, mechanisms not established in this study improved the pathogenicity of the fungal strain of the T4 treatment. In this regard^56–58^ warn that the effect of entomopathogenic fungi depends on the contact of the fungus with the host, the latter being one of the main determinants in the success of IPM methods.

In that sense, the loss of effectiveness *L. lecanii* reaffirm the observations of^56–58^, who warn that fungi are susceptible to solar radiation and depend on the available water to activate, maintain and end their biological cycle. We believe that entomopathogens were differentially affected by the conditions of high radiation and low rainfall during the period of strong summer caused by the phenomenon “El Niño” during June 2015 to April 2016^59^. This duality in the field results of entomopathogenic fungi has been an argument against their use: there is less certainty of their effectiveness than, for example, chemical alternatives^58^.

The results for *S. ampliophilobia*, are revealing: suggest that between commercial biological strategies (spinosad), chemical (S-1,2 di (ethoxycarbonyl) ethyl 0,0-dimethyl phosphorodithioate) and cultural (pruning and silvering), there are no statistical differences that allow one method to be privileged over another, corroborating the growing trend towards a decrease in the gap between pesticides, biopesticides and cultural practices (Bettiol et al.^29^), which, in statistical terms of the effectiveness of *S. ampliophilobia* population management under HRS conditions, would have no contrast (Fig. 3b). Therefore, the choice of the management method would not depend on its effectiveness but on its cost, ease of acquisition, implementation and little harm to human and animal health and the environment. Under all the previous premises, cultural treatment is positioned as the best alternative.

## Conclusions

Although the chemical treatment showed the highest efficiency in laboratory conditions against pests, especially against *C. theobromae*, it was found that the field conditions represented especially in the pruning and keeping the area around the plants clear of weeds as treatment, offer adverse conditions for the survival of pests, as much or even more efficient than biopesticides or chemical control. The foregoing shows that the constant application of pruning, sanitation, hygiene, and sanitary waste management practices in orchards is essential to control these two pests.

This statistical parity of cultural practices, and biological and chemical management is an argument in favor of the use of the former to the detriment of the third, especially when the harmful effects of the molecule S-1,2 di (ethoxycarbonyl) ethyl 0, 0-dimethyl phosphorodithioate have been proven in air, water and agricultural soils, in addition to its association with thyroid cancer in humans. This is a strong argument to favor the use of synergies of cultural and biological management methods framed in IPM, as opposed to the use of chemical agents whose harmful effects are strongly documented, and whose use is becoming increasingly prohibited.

## Supplementary Information

Supplementary Information.
