# Supplementary material for: Management alternatives for Carmenta theobromae (Busck, 1910) (Lepidoptera: Sesiidae) and Simplicivalva ampliophilobia (Lepidoptera: Cossidae), limiting pests of guava in Colombia
Source: Sci Rep. 2021 Feb 4;11:3076. doi: 10.1038/s41598-021-81830-3 (PMC7862407; doi:10.1038/s41598-021-81830-3)
Supplement: Supplementary file 1 — Supplementary Information. [file 41598_2021_81830_MOESM1_ESM.docx]

MANAGEMENT ALTERNATIVES FOR Carmenta theobromae (Busck, 1910) (Lepidoptera: Sesiidae) AND Simplicivalva ampliophilobia (Lepidoptera: Cossidae), LIMITING PESTS OF GUAVA IN COLOMBIA

Running title: MANAGEMENT ALTERNATIVES FOR LIMITING GUAVA PESTS IN COLOMBIA

Víctor Camilo Pulido-Blanco^1^*, Elberth Hernando Pinzón-Sandoval^2^, Carlos Felipe González Chavarro^3^, Pablo Antonio Serrano Cely^4^.

* Corresponding author: vpulido@agrosavia.co; victor.pulido@catie.ac.cr

^1^M.Sc. Ciencias Biológicas, Corporación Colombiana de Investigación Agropecuaria - AGROSAVIA, Centro de Investigación Tibaitata, Kilometro 14 vía Mosquera - Cundinamarca, ORCID: https://orcid.org/0000-0002-1217-6877 / Correspondence details: Calle 19 N° 9-35 Edificio de la Lotería de Boyacá, oficina 902, Tunja, Boyacá, Colombia; Email: vpulido@agrosavia.co; victor.pulido@catie.ac.cr

^2^M.Sc. Fisiología Vegetal. Universidad Pedagógica y Tecnológica de Colombia, Tunja-Colombia. ORCID: http://orcid.org/0000-0001-9229-3450

^3^M.sc. Fisiología Vegetal, Corporación Colombiana de Investigación Agropecuaria - AGROSAVIA, Centro de Investigación La Libertad, Kilometro 17 vía Puerto López - Meta. ORCID: https://orcid.org/0000-0002-1720-5067

^4^M.Sc. Ciencias Ambientales. Universidad Pedagógica y Tecnológica de Colombia, Tunja-Colombia. ORCID: http://orcid.org/0000-0002-1270-3024

**Supplementary material**

**Supplementary table 1.** Body length and body mass of *Carmenta theobromae* larvae under laboratory conditions

| **Larva** | **Length (mm)** | **Mass (g)** | **Observations** |
| --- | --- | --- | --- |
| **1** | 14 | 0.063 | Dead |
| **1** | 18 | 0.049 |  |
| **2** | 10 | 0.034 | Dead |
| **2** | 17 | 0.042 |  |
| **3** | 9 | 0.011 | Dead |
| **3** | 9 | 0.01 |  |
| **4** | 12 | 0.027 | Dead |
| **4** | 16 | 0.054 |  |
| **5** | 14 | 0.059 | Dead |
| **5** | 11 | 0.021 |  |
| **6** | 13 | 0.047 | Dead |
| **6** | 12 | 0.031 |  |
| **7** | 10 | 0.037 | Dead |
| **7** | 14 | 0.041 |  |
| **8** | 13 | 0.048 | Dead |
| **8** | 12 | 0.024 |  |
| **9** | 14 | 0.054 | Dead |
| **9** | 5 | 0.003 |  |
| **10** | 10 | 0.042 | Dead |
| **10** | 5 | 0.003 |  |
| **11** | 5 | 0.007 | Dead |
| **11** | 12 | 0.03 |  |
| **12** | 16 | 0.068 | Dead |
| **12** | 15 | 0.039 |  |
| **13** | 15 | 0.089 | Dead |
| **13** | 9 | 0.012 |  |
| **14** | 13 | 0.041 | Dead |
| **14** | 8 | 0.012 |  |
| **15** | 12 | 0.053 | Dead |
| **15** | 11 | 0.035 |  |
| **16** | 12 | 0.065 | Dead |
| **16** | 15 | 0.04 |  |
| **17** | 7 | 0.011 | Dead |
| **17** | 11 | 0.016 |  |
| **18** | 13 | 0.05 | Dead |
| **18** | 12 | 0.022 |  |
| **19** | 9 | 0.024 | Dead |
| **20** | 15 | 0.075 |  |
| **20** | 13 | 0.035 | Dead |
| **21** | 12 | 0.038 |  |
| **21** | 16 | 0.05 | Dead |
| **22** | 15 | 0.064 |  |
| **22** | 10 | 0.015 | Dead |
| **23** | 15 | 0.071 |  |
| **23** | 7 | 0.008 | Dead |
| **24** | 13 | 0.044 |  |
| **24** | 13 | 0.027 | Dead |
| **25** | 12 | 0.058 |  |
| **25** | 11 | 0.037 | Dead |
| **26** | 7 | 0.012 |  |
| **26** | 16 | 0.051 | Dead |
| **27** | 11 | 0.045 |  |
| **27** | 15 | 0.056 | Dead |
| **28** | 17 | 0.103 |  |
| **28** | 9 | 0.013 | Dead |
| **29** | 10 | 0.029 |  |
| **29** | 8 | 0.038 | Dead |
| **30** | 13 | 0.064 |  |
| **30** | 13 | 0.051 | Dead |
| **31** | 9 | 0.02 |  |
| **32** | 12 | 0.051 | Dead |
| **32** | 7 | 0.008 |  |
| **33** | 15 | 0.071 | Dead |
| **33** | 14 | 0.046 |  |
| **34** | 15 | 0.043 |  |
| **35** | 11 | 0.047 |  |
| **35** | 9 | 0.011 |  |
| **36** | 8 | 0.032 |  |
| **36** | 3 | 0.002 |  |
| **37** | 7 | 0.008 |  |
| **38** | 8 | 0.01 | Dead |
| **38** | 12 | 0.055 |  |
| **39** | 5 | 0.01 | Dead |
| **39** | 11 | 0.013 |  |
| **40** | 9 | 0.013 |  |
| **41** | 12 | 0.025 |  |
| **42** | 11 | 0.024 |  |
| **43** | 14 | 0.063 |  |
| **44** | 4 | 0.005 |  |
| **45** | 15 | 0.075 |  |
| **46** | 11 | 0.019 |  |
| **47** | 18 | 0.117 |  |
| **48** | 16 | 0.081 |  |
| **49** | 12 | 0.076 |  |
| **50** | 13 | 0.029 |  |
| **51** | 14 | 0.047 |  |
| **52** | 15 | 0.041 |  |
| **53** | 9 | 0.014 |  |
| **54** | 18 | 0.089 |  |
| **55** | 18 | 0.071 |  |
| **56** | 5 | 0.002 |  |
| **57** | 15 | 0.076 |  |
| **58** | 18 | 0.065 |  |
| **59** | 11 | 0.015 |  |
| **60** | 12 | 0.026 |  |
| **61** | 15 | 0.036 |  |
| **62** | 12 | 0.025 |  |
| **63** | 9 | 0.01 |  |
| **64** | 12 | 0.025 |  |
| **65** | 5 | 0.001 |  |
| **66** | 4 | 0.001 |  |
| **79** | 7 | 0.007 |  |
| **80** | 17 | 0.078 |  |
| **81** | 14 | 0.072 |  |
| **82** | 9 | 0.011 |  |
| **83** | 15 | 0.055 |  |
| **84** | 8 | 0.026 |  |
| **85** | 7 | 0.006 |  |
| **86** | 11 | 0.02 |  |
| **87** | 11 | 0.022 |  |
| **88** | 15 | 0.063 |  |
| **89** | 15 | 0.072 |  |
| **90** | 13 | 0.055 |  |
| **91** | 12 | 0.045 |  |
| **92** | 10 | 0.04 |  |
| **93** | 10 | 0.034 |  |
| **94** | 16 | 0.089 |  |
| **95** | na | na | Dead |
| **96** | 14 | 0.048 |  |
| **97** | 11 | 0.038 |  |
| **98** | 14 | 0.07 |  |
| **99** | 12 | 0.048 |  |
| **100** | 5 | 0.004 |  |
| **101** | 10 | 0.027 |  |
| **102** | 15 | 0.078 |  |
| **103** | 15 | 0.072 |  |
| **104** | 9 | 0.026 |  |
| **105** | 13 | 0.065 |  |
| **106** | 15 | 0.092 |  |
| **107** | 8 | 0.028 |  |
| **108** | 8 | 0.021 |  |
| **109** | 8 | 0.012 |  |
| **110** | 12 | 0.034 |  |
| **111** | 14 | 0.052 |  |
| **112** | 9 | 0.015 |  |
| **113** | 12 | 0.029 |  |
| **114** | 13 | 0.056 |  |
| **115** | 12 | 0.036 |  |
| **116** | 9 | 0.014 |  |
| **117** | 15 | 0.064 |  |
| **118** | 13 | 0.053 |  |
| **119** | 15 | 0.096 |  |
| **120** | 10 | 0.035 |  |
| **121** | 13 | 0.047 |  |
| **122** | 15 | 0.046 |  |
| **123** | 14 | 0.058 |  |
| **124** | 11 | 0.033 |  |
| **125** | 14 | 0.06 |  |
| **126** | 8 | 0.01 |  |
| **127** | 12 | 0.042 |  |
| **128** | 12 | 0.025 |  |
| **129** | 11 | 0.036 |  |
| **130** | 6 | 0.005 |  |
| **131** | 14 | 0.056 |  |
| **132** | 6 | 0.001 | Dead |
| **133** | 2 | 0.001 | Dead |
| **134** | 12 | 0.053 |  |
| **135** | 9 | 0.023 |  |
| **136** | 5 | 0.001 | Dead |
| **137** | 8 | 0.019 |  |
| **138** | 12 | 0.049 |  |
| **139** | 4 | 0.001 |  |
| **140** | 14 | 0.057 |  |
| **141** | 7 | 0.006 |  |
| **142** | 6 | 0.004 |  |

na: not available

**Supplementary table 2.** Length and body mass of *Carmenta theobromae* larvae under field conditions

| **Larva** | **Length (mm)** | **Mass (g)** |
| --- | --- | --- |
| **1** | 17 | 0.077 |
| **2** | 15 | 0.047 |
| **3** | 7 | 0.009 |
| **4** | 14 | 0.036 |
| **5** | 6 | 0.005 |
| **6** | 12 | 0.032 |
| **7** | 10 | 0.021 |
| **8** | 18 | 0.092 |
| **9** | 17 | 0.081 |
| **10** | 8 | 0.008 |
| **11** | 16 | 0.105 |
| **12** | 11 | 0.026 |
| **13** | 10 | 0.029 |
| **14** | 11 | 0.023 |
| **15** | 8 | 0.005 |
| **16** | 12 | 0.022 |
| **17** | 4 | 0.001 |
| **18** | 6 | 0.004 |
| **19** | 12 | 0.043 |
| **20** | 13 | 0.067 |
| **21** | 5 | 0.002 |
| **22** | 7 | 0.01 |
| **23** | 11 | 0.024 |
| **24** | 6 | 0.004 |
| **25** | 8 | 0.022 |
| **26** | 10 | 0.017 |
| **27** | 15 | 0.066 |
| **28** | 14 | 0.033 |
| **29** | 11 | 0.042 |
| **30** | 4 | 0.001 |
| **31** | 15 | 0.057 |
| **32** | 12 | 0.032 |
| **33** | 17 | 0.059 |
| **34** | 8 | 0.012 |
| **35** | 10 | 0.022 |
| **36** | 11 | 0.015 |
| **37** | 18 | 0.076 |
| **38** | 15 | 0.065 |
| **39** | 12 | 0.048 |
| **40** | 10 | 0.019 |
| **41** | 22 | 0.171 |
| **42** | 11 | 0.026 |
| **43** | 12 | 0.032 |
| **44** | 11 | 0.026 |
| **45** | 6 | 0.002 |
| **46** | 14 | 0.6 |
| **47** | 15 | 0.6 |
| **48** | 8 | 0.008 |
| **49** | 16 | 0.065 |
| **50** | 12 | 0.029 |
| **51** | 13 | 0.053 |
| **52** | 12 | 0.036 |
| **53** | 16 | 0.061 |
| **54** | 6 | 0.005 |
| **55** | 9 | 0.017 |
| **56** | 18 | 0.088 |
| **57** | 16 | 0.074 |
| **58** | 7 | 0.01 |
| **59** | 15 | 0.074 |
| **60** | 16 | 0.073 |
| **61** | 9 | 0.013 |
| **62** | 11 | 0.033 |
| **63** | 6 | 0.004 |
| **64** | 13 | 0.038 |
| **65** | 5 | 0.001 |
| **66** | 5 | 0.001 |
| **67** | 6 | 0.007 |
| **68** | 12 | 0.035 |
| **69** | 16 | 0.097 |
| **70** | 17 | 0.082 |
| **71** | 15 | 0.068 |
| **72** | 15 | 0.058 |
| **73** | 11 | 0.026 |
| **74** | 13 | 0.052 |
| **75** | 18 | 0.097 |
| **76** | 10 | 0.008 |
| **77** | 4 | 0.001 |
| **78** | 0 | 0 |
| **79** | 0 | 0 |
| **80** | 0 | 0 |
| **81** | 0 | 0 |
| **82** | 0 | 0 |
| **83** | 6 | 0.001 |
| **84** | 0 | 0 |
| **85** | 0 | 0 |
| **86** | 18 | 0.049 |
| **87** | 12 | 0.031 |
| **88** | 15 | 0.056 |
| **89** | 9 | 0.02 |
| **90** | 9 | 0.013 |
| **91** | 9 | 0.01 |
| **92** | 12 | 0.055 |
| **93** | 16 | 0.05 |
| **94** | 13 | 0.027 |
| **95** | 14 | 0.063 |
| **96** | 4 | 0.005 |
| **97** | 10 | 0.015 |
| **98** | 13 | 0.029 |
| **99** | 14 | 0.047 |
| **100** | 18 | 0.089 |
| **101** | 15 | 0.076 |

**Supplementary table 3.** Length and body mass of *Simplicivalva ampliaphilobia* larvae under laboratory conditions

| **Larvae** | **Length (mm)** | **Mass (g)** | **Observation** |
| --- | --- | --- | --- |
| **1** | 23.73 | 0.561 |  |
| **2** | 15.09 | 0.096 |  |
| **3** | 0.00 | 0.000 | Dead |
| **4** | 0.00 | 0.000 | Dead |
| **5** | 0.00 | 0.000 | Dead |
| **6** | 0.00 | 0.000 | Dead |
| **7** | 0.00 | 0.000 | Dead |
| **8** | 0.00 | 0.000 | Dead |
| **9** | 0.00 | 0.000 | Dead |
| **10** | 0.00 | 0.000 | Dead |
| **11** | 10.02 | 0.053 |  |
| **12** | 0.00 | 0.000 | Dead |
| **13** | 0.00 | 0.000 | Dead |
| **14** | 0.00 | 0.000 | Dead |
| **15** | 0.00 | 0.000 | Dead |
| **16** | 15.96 | 0.141 |  |
| **17** | 0.00 | 0.000 | Dead |
| **18** | 0.00 | 0.000 | Dead |
| **19** | 0.00 | 0.000 | Dead |
| **20** | 0.00 | 0.000 | Dead |
| **21** | 0.00 | 0.000 | Dead |
| **22** | 0.00 | 0.000 | Dead |
| **23** | 0.00 | 0.000 | Dead |
| **24** | 0.00 | 0.000 | Dead |
| **25** | 0.00 | 0.000 | Dead |
| **26** | 20.08 | 0.324 |  |
| **27** | 0.00 | 0.000 | Dead |
| **28** | 0.00 | 0.000 | Dead |
| **29** | 19.20 | 0.266 |  |
| **30** | 0.00 | 0.000 | Dead |
| **31** | 0.00 | 0.000 | Dead |
| **32** | 0.00 | 0.000 | Dead |
| **33** | 0.00 | 0.000 | Dead |
| **34** | 0.00 | 0.000 | Dead |
| **35** | 22.40 | 0.178 |  |
| **36** | 0.00 | 0.000 | Dead |
| **37** | 0.00 | 0.000 | Dead |
| **38** | 0.00 | 0.000 | Dead |
| **39** | 0.00 | 0.000 | Dead |
| **40** | 0.00 | 0.000 | Dead |
| **41** | 16.22 | 0.117 |  |
| **42** | 0.00 | 0.000 | Dead |
| **43** | 31.32 | 0.592 |  |
| **44** | 20.28 | 0.287 |  |
| **45** | 25.49 | 0.478 |  |
| **46** | 0.00 | 0.000 | Dead |
| **47** | 0.00 | 0.000 | Pupa |
| **48** | 0.00 | 0.000 | Dead |
| **49** | 0.00 | 0.000 | Dead |
| **50** | 24.36 | 0.410 |  |
| **51** | 0.00 | 0.000 | Dead |
| **52** | 0.00 | 0.000 | Dead |
| **53** | 0.00 | 0.000 | Dead |
| **54** | 17.48 | 0.275 |  |
| **55** | 20.67 | 0.199 |  |
| **56** | 22.75 | 0.264 |  |
| **57** | 0.00 | 0.000 | Dead |
| **58** | 25.17 | 0.373 |  |
| **59** | 23.92 | 0.447 |  |
| **60** | 0.00 | 0.000 | Dead |
| **61** | 20.95 | 0.309 |  |
| **62** | 21.42 | 0.275 |  |
| **63** | 21.02 | 0.302 |  |
| **64** | 12.05 | 0.170 |  |
| **65** | 29.63 | 0.713 |  |
| **66** | 23.56 | 0.353 |  |
| **67** | 27.13 | 0.554 |  |
| **68** | 20.20 | 0.212 |  |
| **69** | 24.45 | 0.385 |  |
| **70** | 20.02 | 0.300 | Dead |
| **71** | 0.00 | 0.000 | Dead |
| **72** | 20.91 | 0.262 |  |
| **73** | 0.00 | 0.000 | Dead |
| **74** | 20.82 | 0.253 |  |
| **75** | 23.02 | 0.251 |  |
| **76** | 19.10 | 0.219 |  |
| **77** | 16.65 | 0.117 |  |
| **78** | 26.36 | 0.509 |  |
| **79** | 0.00 | 0.000 | Dead |
| **80** | 14.02 | 0.207 |  |
| **81** | 26.63 | 0.312 |  |
| **82** | 17.08 | 0.187 |  |
| **83** | 29.42 | 0.676 |  |
| **84** | 19.80 | 0.193 |  |
| **85** | 26.91 | 0.460 |  |
| **86** | 30.35 | 0.691 |  |
| **87** | 15.86 | 0.088 |  |
| **88** | 41.13 | 1.201 |  |
| **89** | 21.84 | 0.205 |  |
| **90** | 23.84 | 0.305 |  |
| **91** | 22.84 | 0.270 |  |
| **92** | 17.46 | 0.160 |  |
| **93** | 0.00 | 0.000 | Dead |
| **94** | 22.87 | 0.304 |  |
| **95** | 33.26 | 0.666 |  |
| **96** | 16.05 | 0.010 |  |
| **97** | 18.77 | 0.138 |  |
| **98** | 10.07 | 0.064 |  |
| **99** | 24.45 | 0.330 |  |
| **100** | 0.00 | 0.000 | Dead |
| **101** | 36.94 | 1.295 |  |
| **102** | 37.70 | 0.996 |  |
| **103** | 22.07 | 0.156 |  |
| **104** | 20.39 | 0.233 |  |
| **105** | 18.05 | 0.150 |  |
| **106** | 19.80 | 0.155 |  |
| **107** | 33.68 | 1.152 |  |
| **108** | 23.10 | 0.333 |  |
| **109** | 19.70 | 0.159 |  |
| **110** | 23.51 | 0.338 |  |
| **111** | 31.78 | 0.856 |  |
| **112** | 30.85 | 0.576 |  |
| **113** | 27.75 | 0.497 |  |
| **114** | 23.98 | 0.464 |  |
| **115** | 19.96 | 0.205 |  |
| **116** | 22.16 | 0.264 |  |
| **117** | 23.39 | 0.540 |  |
| **118** | 30.94 | 0.682 |  |
| **119** | 0.00 | 0.000 | Dead |
| **120** | 27.14 | 0.701 |  |
| **121** | 32.55 | 0.809 |  |
| **122** | 32.72 | 0.866 |  |
| **123** | 26.93 | 0.532 |  |
| **124** | 27.80 | 0.626 |  |
| **125** | 24.32 | 0.299 |  |
| **126** | 30.75 | 0.787 |  |
| **127** | 24.80 | 0.406 |  |
| **128** | 28.95 | 0.561 |  |
| **129** | 21.22 | 0.240 |  |
| **130** | 25.82 | 0.362 |  |
| **131** | 25.02 | 0.223 |  |
| **132** | 0.00 | 0.000 | Dead |
| **133** | 24.45 | 0.436 |  |
| **134** | 28.30 | 0.423 |  |
| **135** | 29.33 | 0.569 |  |
| **136** | 24.60 | 0.360 |  |
| **137** | 21.56 | 0.187 |  |
| **138** | 19.42 | 0.127 |  |
| **139** | 34.61 | 1.130 |  |
| **140** | 21.29 | 0.235 |  |
| **141** | 21.26 | 0.210 |  |
| **142** | 19.54 | 0.218 |  |
| **143** | 28.18 | 0.510 |  |
| **144** | 23.61 | 0.499 |  |

**Supplementary table 4.** Length and body mass of larvae of *Simplicivalva ampliaphilobia* in the field

| **Re-tagged** | **Length (mm)** | **Mass (g)** |
| --- | --- | --- |
| **0** | 7 | 0.065 |
| **1** | 10 | 0.04 |
| **2** | 11 | 0.074 |
| **3** | 11 | 0.052 |
| **4** | 11 | 0.046 |
| **5** | 11 | 0.06 |
| **6** | 11 | 0.044 |
| **7** | 11 | 0.052 |
| **8** | 11 | 0.059 |
| **9** | 12 | 0.148 |
| **10** | 12 | 0.127 |
| **11** | 12 | 0.061 |
| **12** | 12 | 0.058 |
| **13** | 12 | 0.081 |
| **14** | 12 | 0.055 |
| **15** | 12 | 0.11 |
| **16** | 13 | 0.138 |
| **17** | 13 | 0.081 |
| **18** | 14 | 0.06 |
| **19** | 15 | 0.113 |
| **20** | 15 | 0.13 |
| **21** | 15 | 0.096 |
| **22** | 15 | 0.108 |
| **23** | 15 | 0.103 |
| **24** | 16 | 0.089 |
| **25** | 17 | 0.11 |
| **26** | 18 | 0.119 |
| **27** | 18 | 0.094 |
| **28** | 18 | 0.166 |
| **29** | 19 | 0.113 |
| **30** | 19 | 0.266 |
| **31** | 19 | 0.096 |
| **32** | 19 | 0.201 |
| **33** | 19 | 0.182 |
| **34** | 20 | 0.195 |
| **35** | 20 | 0.229 |
| **36** | 20 | 0.179 |
| **37** | 20 | 0.141 |
| **38** | 20 | 0.249 |
| **39** | 20 | 0.172 |
| **40** | 20 | 0.209 |
| **41** | 21 | 0.348 |
| **42** | 21 | 0.167 |
| **43** | 22 | 0.255 |
| **44** | 22 | 0.294 |
| **45** | 22 | 0.239 |
| **46** | 23 | 0.293 |
| **47** | 24 | 0.214 |
| **48** | 24 | 0.313 |
| **49** | 25 | 0.382 |
| **50** | 25 | 0.385 |
| **51** | 25 | 0.325 |
| **52** | 28 | 0.427 |
| **53** | 28 | 0.391 |
| **54** | 28 | 0.335 |
| **55** | 28 | 0.683 |
| **56** | 30 | 0.547 |
| **57** | 30 | 0.406 |
| **58** | 30 | 0.441 |
| **59** | 35 | 0.834 |
| **60** | 55 | 0.25 |

**Supplementary table 5.** Distribution of larvae in the study treatments for the management alternatives of *Carmenta theobromae* under laboratory conditions.

| R | Spinosad (a mixture of Spinosad A and D) | S-1,2-di (ethoxycarbonyl) ethyl 0,0-dimethyl phosphorodithioate | Mix of *B. bassiana* and *B. brongniartii* | *Lecanicillium lecanii* | *B. bassiana* | W |
| --- | --- | --- | --- | --- | --- | --- |
| 1 | 80 | 117 | 83 | 122 | 103 | 94 |
|  | 123 | 96 | 111 | 140 | 131 | 90 |
|  | 118 | 92 | 105 | 93 | 99 | 110 |
|  | 124 | 130 | 85 | 87 | 97 | 129 |
|  | 79 | 107 | 108 | 109 | 141 | 104 |
|  |  |  |  |  |  |  |
| 2 | 119 | 88 | 106 | 102 | 89 | 81 |
|  | 121 | 125 | 98 | 114 | 113 | 91 |
|  | 115 | 127 | 134 | 128 | 86 | 138 |
|  | 120 | 116 | 135 | 100 | 142 | 101 |
|  | 84 | 82 | 112 | 137 | 139 | 126 |

R: repetition. W: distilled water (control)

Source: Elaborated by the authors.

**Supplementary table 6.** Distribution of larvae in the study treatments for the management alternatives of *Carmenta theobromae* under field conditions

| R | W | S-1,2-di (ethoxycarbonyl) ethyl 0,0-dimethyl phosphorodithioate | *Lecanicillium lecanii* | *B. bassiana* | C |
| --- | --- | --- | --- | --- | --- |
| 1 | 66 | 63 | 83 | 24 | 21 |
|  | 35 | 16 | 14 | 23 | 12 |
|  | 101 | 57 | 37 | 1 | 9 |
| 2 | 76 | 48 | 10 | 67 | 15 |
|  | 93 | 74 | 86 | 39 | 2 |
|  | 51 | 92 | 88 | 31 | 72 |
| 3 | 26 | 40 | 89 | 7 | 25 |
|  | 28 | 43 | 68 | 62 | 52 |
|  | 71 | 60 | 59 | 20 | 27 |
| 4 | 54 | 45 | 96 | 18 | 5 |
|  | 42 | 44 | 73 | 94 | 13 |
|  | 8 | 100 | 70 | 56 | 69 |
| 5 | 3 | 22 | 58 | 91 | 34 |
|  | 99 | 64 | 4 | 19 | 29 |
|  | 11 | 75 | 46 | 47 | 41 |
| 6 | 55 | 61 | 90 | 36 | 97 |
|  | 50 | 87 | 98 | 6 | 32 |
|  | 33 | 53 | 95 | 38 | 49 |

R: repetition. W: distilled water (control). C: cultural practices that include pruning and keeping the area around the plants clear of weeds

Source: Elaborated by the author.

Supplementary Table 7. Distribution of the larvae in the study treatments of management alternatives of *Simplicivalva ampliaphilobia* in the laboratory.

| R | Spinosad (a mixture of Spinosad A and D) | S-1,2-di(ethoxycarbonyl) ethyl 0,0-dimethyl phosphorodithioate | Mix of *B. bassiana* and *B. brongniartii* | *Lecanicillium lecanii* | *B. bassiana* |
| --- | --- | --- | --- | --- | --- |
| 1 | 87 | 16 | 41 | 92 | 105 |
|  | 50 | 99 | 133 | 136 | 127 |
|  | 131 | 58 | 85 | 123 | 120 |
| 2 | 97 | 76 | 29 | 138 | 142 |
|  | 144 | 1 | 90 | 114 | 125 |
|  | 113 | 143 | 134 | 135 | 65 |
| 3 | 109 | 106 | 115 | 26 | 68 |
|  | 94 | 75 | 108 | 117 | 66 |
|  | 126 | 112 | 118 | 111 | 121 |
| 4 | 104 | 55 | 74 | 63 | 129 |
|  | 141 | 140 | 137 | 103 | 56 |
|  | 122 | 107 | 139 | 101 | 88 |

R: repetition

Source: elaborated by the author.

**Supplementary table 8.** Distribution of the larvae in the study treatments of management alternatives of *Simplicivalva ampliaphilobia* under field conditions.

| R | Spinosad (mixture of Spinosad A and D) | S-1,2-di (ethoxycarbonyl) ethyl 0,0-dimethyl phosphorodithioate | Mix of *B. bassiana* and *B. brongniartii* | C | Distilled water (control) |
| --- | --- | --- | --- | --- | --- |
| 1 | 68 | 62 | 69 | 76 | 78 |
|  | 71 | 6 | 115 | 105 | 98 |
|  | 82 | 95 | 101 | 103 | 113 |
| 2 | 20 | 18 | 77 | 121 | 99 |
|  | 36 | 70 | 102 | 59 | 79 |
|  | 64 | 63 | 111 | 38 | 11 |
| 3 | 66 | 74 | 109 | 8 | 28 |
|  | 35 | 67 | 94 | 96 | 97 |
|  | 114 | 106 | 100 | 119 | 110 |
| 4 | 75 | 58 | 104 | 1 | 3 |
|  | 73 | 34 | 72 | 107 | 118 |
|  | 49 | 116 | 120 | 108 | 22 |

R: repetition. C: cultural practices that include pruning and keeping the area around the plants clear of weeds

Source: elaborated by the author.

| **A.**   \| **ANOVA** \| \| \| \| \| \| \| --- \| --- \| --- \| --- \| --- \| --- \| \| **Dead_larvae** \| \| \| \| \| \| \|  \| Sum of squares \| df \| Square root \| F \| Sig. \| \| Inter-groups \| 69.204 \| 5 \| 13.841 \| 14.727 \| .000 \| \| Intra-groups \| 45.111 \| 48 \| .940 \|  \|  \| \| Total \| 114.315 \| 53 \|  \|  \|  \|  \| **Dead_larvae** \| \| \| \| \| \| --- \| --- \| --- \| --- \| --- \| \| Tukey’s HSD^a^ \| \| \| \| \| \| Study Treatments \| N \| Subset for alpha = 0.05 \| \| \| \| 1 \| 2 \| 3 \| \| Spinosad \| 9 \| .67 \|  \|  \| \| Mix of *B. bassiana* and *B. brongniartii* (powder) \| 9 \| .67 \|  \|  \| \| Distilled water (control) \| 9 \| .78 \|  \|  \| \| *Beauveria bassiana* (liquid) \| 9 \| 1.67 \| 1.67 \|  \| \| *Lecanicillium lecanii* \| 9 \|  \| 2.33 \|  \| \| S-1,2-di(ethoxycarbonyl) ethyl 0,0-dimethyl phosphorodithioate \| 9 \|  \|  \| 3.78 \| \| S.I.G. \|  \| .262 \| .691 \| 1.000 \| \| The means for the groups in the homogeneous subsets are shown. \| \| \| \| \| \| 1. Use the sample size of the harmonic mean = 9,000. \| \| \| \| \| |
| --- | --- | --- | --- | --- | --- | --- | --- | --- | --- | --- | --- | --- | --- | --- | --- | --- | --- | --- | --- | --- | --- | --- | --- | --- | --- | --- | --- | --- | --- | --- | --- | --- | --- | --- | --- | --- | --- | --- | --- | --- | --- | --- | --- | --- | --- | --- | --- | --- | --- | --- | --- | --- | --- | --- | --- | --- | --- | --- | --- | --- | --- | --- | --- | --- | --- | --- | --- | --- | --- | --- | --- | --- | --- | --- | --- | --- | --- | --- | --- | --- | --- | --- | --- | --- | --- | --- | --- | --- | --- | --- | --- | --- | --- | --- | --- | --- | --- | --- | --- |
| **B.**   \| **ANOVA Alternatives for the management of *S. ampliophilobia* under laboratory conditions** \| \| \| \| \| \| \| --- \| --- \| --- \| --- \| --- \| --- \| \| **Average_dead_larvae** \| \| \| \| \| \| \|  \| Sum of squares \| df \| Sum of squares \| F \| Sig. \| \| Inter-groups \| 19.956 \| 5 \| 3.991 \| 43.266 \| .000 \| \| Intra-groups \| 1.660 \| 18 \| .092 \|  \|  \| \| Total \| 21.617 \| 23 \|  \|  \|  \|  \| **Average_dead_larvae** \| \| \| \| \| \| --- \| --- \| --- \| --- \| --- \| \| Tukey’s HSD^a^ \| \| \| \| \| \| Study Treatments \| N \| Subset for alpha = 0.05 \| \| \| \| 1 \| 2 \| 3 \| \| Distilled water (control) \| 4 \| .0000000 \|  \|  \| \| *Beauveria bassiana* (liquid) \| 4 \|  \| 1.9011628 \|  \| \| *Lecanicillium lecanii* \| 4 \|  \| 2.0581395 \| 2.0581395 \| \| Mix of *B. bassiana* and *B. brongniartii* (powder) \| 4 \|  \| 2.1686047 \| 2.1686047 \| \| S-1,2-di(ethoxycarbonyl) ethyl 0,0-dimethyl phosphorodithioate \| 4 \|  \|  \| 2.6627907 \| \| Spinosad \| 4 \|  \|  \| 2.7383721 \| \| S.I.G. \|  \| 1.000 \| .809 \| .051 \| \| The means for the groups in the homogeneous subsets are shown. \| \| \| \| \| \| a. Use the sample size of the harmonic mean = 4,000. \| \| \| \| \| |

**Supplementary figure 1.** Statistics on: A. *C. theobromae* and B. *S. ampliophilobia* under laboratory conditions.

| **A.**   \| **ANOVA** \| \| \| \| \| \| \| --- \| --- \| --- \| --- \| --- \| --- \| \| **Average_dead_larvae** \| \| \| \| \| \| \|  \| Sum of squares \| df \| Square root \| F \| Sig. \| \| Inter-groups \| 3.467 \| 4 \| .867 \| 3.939 \| .013 \| \| Intra-groups \| 5.500 \| 25 \| .220 \|  \|  \| \| Total \| 8.967 \| 29 \|  \|  \|  \|  \| **Average_dead_larvae** \| \| \| \| \| --- \| --- \| --- \| --- \| \| Tukey’s HSD^a^ \| \| \| \| \| Study Treatments \| N \| Subset for alpha = 0.05 \| \| \| 1 \| 2 \| \| *Lecanicillium lecanii* \| 6 \| 2,17 \|  \| \| Distilled water (control) \| 6 \| 2,33 \| 2,33 \| \| S-1,2-di(ethoxycarbonyl) ethyl 0,0-dimethyl phosphorodithioate \| 6 \| 2,67 \| 2,67 \| \| *Beauveria bassiana* (liquid) \| 6 \|  \| 3,00 \| \| Pruning and keeping the area around the plants clear of weeds \| 6 \|  \| 3,00 \| \| S.I.G. \|  \| ,371 \| ,132 \| \| The means for the groups in the homogeneous subsets are shown. \| \| \| \| \| a. Use the sample size of the harmonic mean = 6,000. \| \| \| \| |
| --- | --- | --- | --- | --- | --- | --- | --- | --- | --- | --- | --- | --- | --- | --- | --- | --- | --- | --- | --- | --- | --- | --- | --- | --- | --- | --- | --- | --- | --- | --- | --- | --- | --- | --- | --- | --- | --- | --- | --- | --- | --- | --- | --- | --- | --- | --- | --- | --- | --- | --- | --- | --- | --- | --- | --- | --- | --- | --- | --- | --- | --- | --- | --- | --- | --- | --- | --- | --- | --- | --- | --- | --- | --- | --- | --- | --- | --- | --- | --- | --- | --- | --- |
| **B.**   \| **ANOVA** \| \| \| \| \| \| \| --- \| --- \| --- \| --- \| --- \| --- \| \| **Dead_*S.ampliophilobia*_larvae** \| \| \| \| \| \| \|  \| Sum of squares \| df \| Square root \| F \| Sig. \| \| Square root Inter-group \| 30,264 \| 4 \| 7,566 \| 7,821 \| ,000 \| \| Intra-groups \| 101,580 \| 105 \| ,967 \|  \|  \| \| Total \| 131,843 \| 109 \|  \|  \|  \|  \| **Dead_*S. ampliophilobia*_larvae** \| \| \| \| \| \| --- \| --- \| --- \| --- \| --- \| \| Tukey’s HSD^a^ \| \| \| \| \| \| Management_alternative \| N \| Subset for alpha = 0.05 \| \| \| \| 1 \| 2 \| 3 \| \| Distilled water \| 22 \| .8182 \|  \|  \| \| Mix of *Beauveria bassiana* and *B. brongniartii* \| 22 \| 1.2500 \| 1.2500 \|  \| \| Pruning and keeping the area around the plants clear of weeds \| 22 \| 1.5455 \| 1.5455 \| 1.5455 \| \| S-1,2-di(ethoxycarbonyl) ethyl 0,0-dimethyl phosphorodithioate \| 22 \|  \| 1.9545 \| 1.9545 \| \| Spinosad \| 22 \|  \|  \| 2.3182 \| \| S.I.G. \|  \| .110 \| .130 \| .077 \| \| The means for the groups in the homogeneous subsets are shown. \| \| \| \| \| \| a. Use the sample size of the harmonic mean = 22,000. \| \| \| \| \| |

**Supplementary figure 2.** Statistics of: A. *C. theobromae* and B*.* *S. ampliophilobia* under field conditions
